# Cluster sets and traditional sets elicit similar muscular hypertrophy: a volume and effort-matched study in resistance-trained individuals

**DOI:** 10.1007/s00421-025-05712-6

**Published:** 2025-02-11

**Authors:** Salvador Vargas-Molina, Manuel García-Sillero, Sergio Maroto-Izquierdo, Eneko Baz-Valle, Borja Bautista-Mayorga, Mora Murri, Brad J. Schoenfeld, Javier Benítez-Porres

**Affiliations:** 1 Physical Education and Sport, Faculty of Medicine, University of Málaga, Málaga, Spain; 2 Research Division, Dynamical Business and Science Society–DBSS International SAS, Bogotá, Colombia; 3 i+HeALTH, European University Miguel de Cervantes, Valladolid, Spain; 4 Department of Physical Education and Sport, University of the Basque Country UPV/EHU Vitoria-Gasteiz, Vitoria-Gasteiz, Spain; 5 EADE-University of Wales Trinity Saint David, Málaga, Spain; 6 Endocrinology and Nutrition Clinical Management Unit, Hospital Clínico Virgen de la Victoria; Instituto de Investigación Biomédica de Málaga y Plataforma en Nanomedicina-IBIMA Plataforma BIONAND, Málaga, Spain; 7 Department of Cardiology and Cardiovascular Surgery, Hospital Universitario Virgen de la Victoria, Málaga, Spain; 8 CIBER Fisiopatología de la Obesidad y Nutrición (CIBEROBN), Instituto de Salud Carlos III, Málaga, Spain; 9 Department of Exercise Science and Recreation, CUNY Lehman College, Bronx, NY USA; 10 Internal Medicine UGC, Hospital Regional Universitario de Málaga; Instituto de Investigación Biomédica de Málaga y Plataforma en Nanomedicina-IBIMA Plataforma BIONAND, Málaga, Spain

**Keywords:** Rest-pause training, Hypertrophy, Muscle-mass, Drop sets, Body composition, Body building

## Abstract

**Background and Objective:**

Previous studies examining the effects of cluster sets (CS) compared to traditional sets (TS) protocols on muscle hypertrophy have primarily equated to volume load. This inevitably has resulted in a lower number of repetitions performed in TS compared to CS, thereby leading to a suboptimal hypertrophic stimulus. The present study aimed to compare the impact of CS and TS protocols, both performed with the same number of sets and repetitions, but with loads adjusted to the same range of repetitions in reserve (RIR) on muscle hypertrophy.

**Methods:**

Ten resistance-trained volunteers (7 men and 3 women, 21.0 ± 1.5 years, 64.3 ± 6.9 kg, and 169.3 ± 6.2 cm) participated in this study. Participants performed two training protocols over an 8-week period, with two weekly sessions consisting of 5 sets of 12 repetitions of the leg press and leg extension exercises. The study employed a within-participant, unilateral design where one limb performed a TS protocol and the contralateral limb performed 3 clusters of 4 repetitions with a 20-s intra-set rest period of the same exercises (CS). Muscle thickness was assessed via ultrasound and thigh lean tissue mass was assessed by dual-energy X-ray absorptiometry pre- and post-study.

**Results:**

Results showed similar increases in muscle thickness (*p* < 0.001, ES = 0.56, and *p* = 0.012, ES = 0.42, respectively) and lean tissue mass (*p* = 0.002, ES = 0.11, and *p* < 0.001, ES = 0.13, respectively) in both CS and TS conditions.

**Conclusion:**

In conclusion, when sets, repetitions, and load adjustments were equalized based on RIR, a CS protocol elicits similar increases in muscle thickness and lean mass compared to a TS protocol.

**Supplementary Information:**

The online version contains supplementary material available at 10.1007/s00421-025-05712-6.

## Introduction

Cluster sets (CS) are an alternative to traditional strength training that involves partitioning a conventional set into repetition blocks, with short intra-set rest periods interspersed between clusters (Tufano et al. 2017a; Nagatani et al. 2022). Current research has demonstrated the positive effects of CS for optimizing neuromuscular and sports performance, including improvements in jump height and sprint capacity (Moreno et al. 2014; Asadi and Ramirez-Campillo 2016), and enhancements in strength and mechanical power (Morales-Artacho et al. 2018). In addition, CS have been shown to improve maximum velocity and power output during weightlifting (Tufano et al. 2017b).

CS also have been proposed as a strategy to promote muscle hypertrophy (Vargas-Molina et al. 2021; Erick Carlos da Cunha Totó et al. 2019). There is indirect evidence supporting its effectiveness in this regard, such as increased total repetition volume (Iglesias-Soler et al. 2014) and reduced concentric velocity loses during weightlifting (Garcia-Ramos et al. 2020) leading to greater mechanical responses with a higher total volume (Oliver et al. 2016). However, only nine studies to date have longitudinally examined the impact of CS on measures of muscle hypertrophy. When anthropometric measurements were used to compare the two methodologies, no differences in muscle circumference increases were observed between protocols (Arazi et al. 2018; Iglesias-Soler et al. 2016; Carneiro et al. 2019). However, this may be due to the low sensitivity of the measurement technique. Conversely, when lean mass was measured using dual-energy X-ray absorptiometry (DXA), increases in lean mass were found following the implementation of CS (Oliver et al. 2013; Bonilla et al. 2021). However, it should be noted that lean tissue mass is not a direct measure of hypertrophy, which may obscure the ability to draw inferences on changes in muscle mass between CS and traditional resistance training protocols.

Magnetic resonance imaging (MRI) is considered the gold-standard for directly measuring muscle mass (Zhao et al. 2013). B-mode ultrasound has been shown to be a viable, cost-effective alternative to MRI for the assessment of skeletal muscle hypertrophy (Franchi et al. 2018). When comparing the increase in muscle size via ultrasound, evidence indicates that TS results in greater changes in muscle thickness compared to CS in both the lower (Goto et al. 2005) and upper limbs (Davies et al. 2022). These findings have cast doubt as to the efficacy of CS for the purpose of optimizing muscle development. However, the studies in question matched the total volume (in terms of the number of sets and repetitions) and intensity (i.e., % of 1-RM) used in both CS and TS protocols. This implies that the load used in CS protocols results in a lower number of repetitions performed compared to the achievable repetitions (i.e., deviating from the repetition in reserve [RIR] 0–4 range, which is more conducive to muscle hypertrophy) (Schoenfeld and Grgic 2019a, b). Therefore, it remains undetermined whether a CS protocol that equates the total number of sets and repetitions but adjusts the intensity within each cluster to a RIR of 0 to 1, is equally effective, or perhaps more so, as traditional training.

Due to the paucity of evidence comparing CS and TS protocols with load adjustments based on RIR, as well as the conflicting empirical evidence regarding the effect of CS on muscle hypertrophy, the purpose of this study was to compare increases in lean mass and muscle thickness following CS and TS protocols, while keeping the total number of repetitions and sets the same but adjusting the intensity of each cluster and each set in TS to a RIR of 0–1.

Our hypothesis is that cluster training, compared to traditional training, leads to a higher volume load (calculated as sets × repetitions × load in kg) when the number of sets and repetitions are matched. This is attributed to the higher intensity achievable when prescribing 4 repetitions compared to 12 repetitions, particularly when intensity is based on repetitions in reserve. Conceivably, the greater volume load accumulated by the CS group would be expected to lead to greater gains in muscle hypertrophy. We hypothesized that by matching total volume and effort levels (same number of RIR), CS would elicit greater hypertrophy than TS.

## Methods

### Participants

**Fig. 1:**
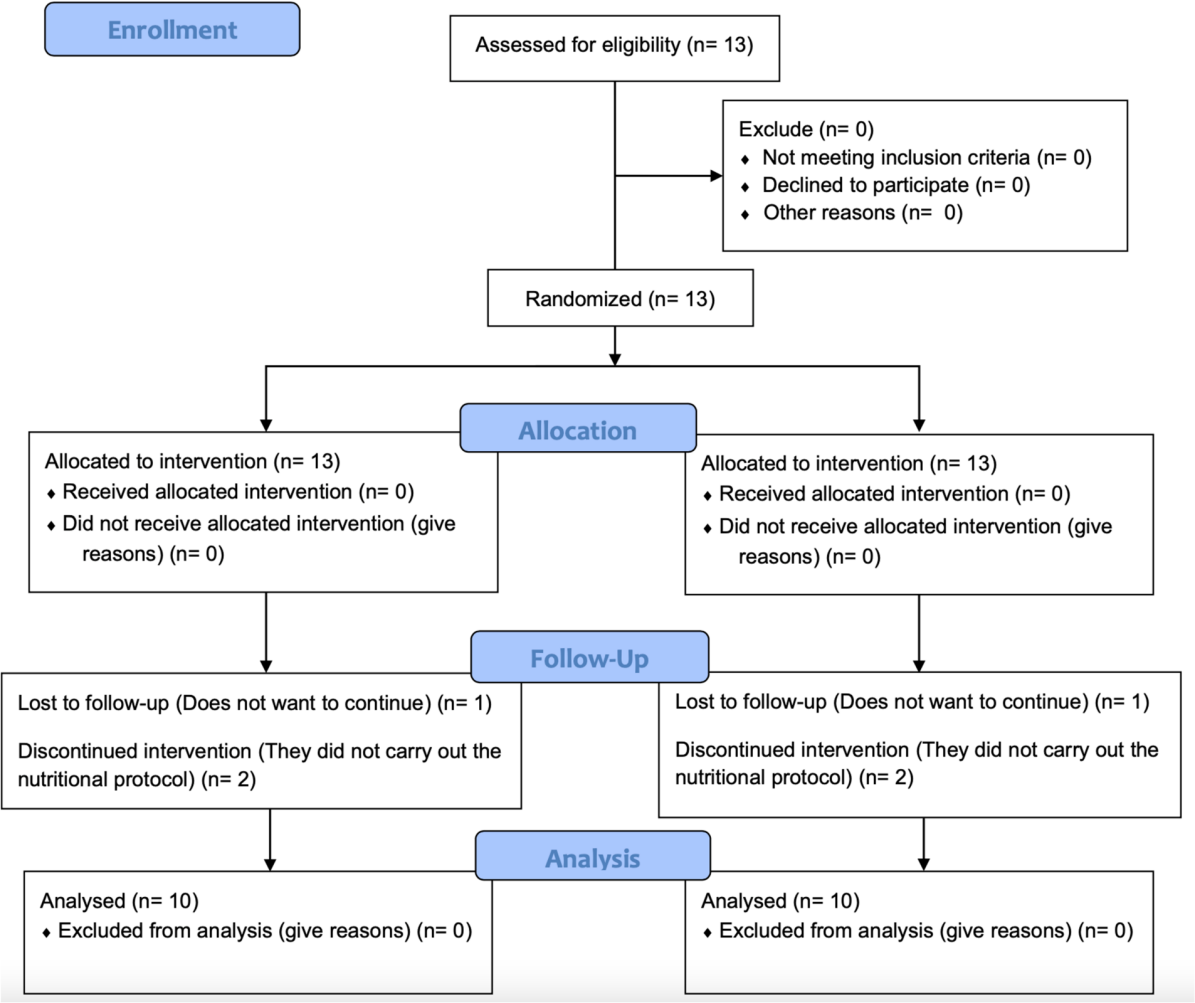
Consolidated standards of reporting trials (CONSORT) flow diagram

Thirteen participants with a minimum of two years of continuous experience in RT performed 3 to 5 sessions per week, including training for all major muscle groups volunteered to participate in this study (mean ± SD, 21.0 ± 1.5 years, 64.3 ± 6.9 kg, 169.3 ± 6.2 cm). All individuals agreed to adhere to the prescribed training regimen for the duration of the 8-week study period and refrain from engaging in any additional physical activity. To qualify for participation, potential participants had to be between the age range of 18 to 35 years and self-report to be free of the use of exogenous doping substances (such as anabolic androgenic steroids) within the past 2 years. Prior to enrollment, participants were provided with information regarding the potential risks involved in the experiment and gave their written informed consent to participate. The research protocol received approval from the Ethics Committee of the University of Malaga (code: 38-2019-H) in accordance with the ethical guidelines outlined in the Declaration of Helsinki (WMA 2013). The enrollment process and participant exclusions illustrated in Fig. 1, following the guidelines of the Consolidated Standards of Reporting Trials (CONSORT) diagram.

### Procedure

#### Training protocols

**Fig. 2:**
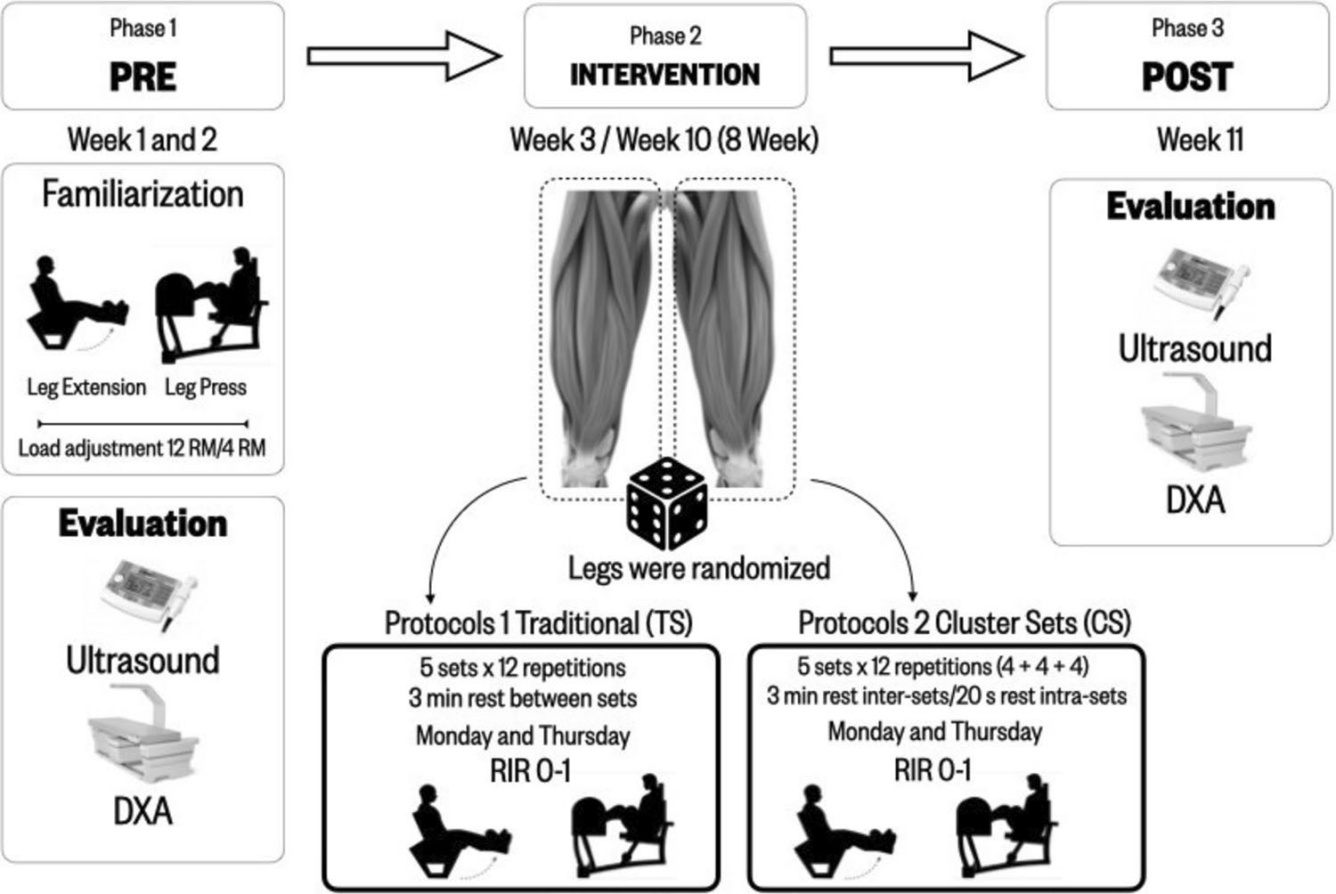
Visual diagram of the experimental design and procedures

We employed a within-subject design whereby participants served as their own control, reducing intra-individual variability and thus enhancing statistical power (MacInnis et al. 2017). Participants performed two weekly training sessions using the leg extension (LE) and leg press (LP) exercises (Gervasport, Madrid, Spain) unilaterally, with both legs trained during each session. The CS and TS protocols were randomly assigned to each participants’ lower limb using an online application (http://www.randomizer.org). To avoid the potential influence of limb dominance on the results, we randomized which leg (dominant or non-dominant) performed each protocol. This approach ensured an equal distribution of participants completing each protocol with either their dominant or non-dominant leg. Each protocol involved 5 sets of 12 repetitions. The CS protocol included 3 clusters of 4 repetitions, with a 20-s intra-set rest based on previous findings from our laboratory (Vargas-Molina et al. 2020). A 3-min recovery period was provided between sets. All training sessions were closely supervised by two researchers, ensuring that the loads were individually tailored for each leg, participant, and session. The loads for each protocol were adjusted to reach failure or be within a maximum of one repetition from failure (i.e., repetitions in reserve [RIR] of 0–1). This load adjustment was applied to both the TS protocol, with 12 repetitions per set, and the CS protocol, with 4 repetitions per block. When the load lifted during any of the protocols allowed for more than two or three repetitions remaining in the tank (RIR of 2–3), the load was increased for the subsequent sets. Participants determined their RIR at the end of each set and loads were then maintained, reduced or increased in subsequent sets according to their perceived effort. Similarly, if on a particular day or set, the prescribed number of repetitions (12 or 4) was not achieved according to the protocol, a short rest period was taken, and the remaining repetitions were completed. If this occurred and there were still sets remaining, the load was reduced for the next set. Throughout the study, all participants performed the designated 5 sets of 12 repetitions. Additionally, participants were instructed to perform repetitions with a velocity of approximately one second on both concentric and eccentric actions to control for time under tension. This variable was monitored by both researchers at each training session. Figure 2 presents a detailed illustration of the protocols. Training volume was calculated individually for each participant, exercise, condition, and session by multiplying the average load used per session by the number of sets and repetitions.

#### Dietary Intake

Participants were instructed to consume 2 g·kg^−1^·d^−1^ of protein to optimize the increase in fat-free mass (FFM) (Jager et al. 2017), 1 g·kg^−1^·d^−1^ of dietary fat and the remaining calories in the form of carbohydrates, totaling 45 kcal kg^−1^·d^−1^ of FFM (Romance et al. 2019). An individualized diet plan was prescribed for each participant, and they were required to track their food intake and maintain a nutritional record for the duration of the program using the MyFitnessPal application (MyFitnessPal, LLC, CA, USA) (Teixeira et al. 2018). Weekly monitoring of food consumption was conducted to ensure adherence to the prescribed dietary regimen.

### Measures

#### Dual-energy x-ray absorptiometry

Participants were instructed to arrive at the lab having fasted for at least 12 h. Prior to testing, participants voided their bladder. Additionally, all the evaluations were conducted on a Monday, when the participants had not trained for at least two days.

Total body mass and regional body composition were estimated using dual X-ray absorptiometry (DXA) (APEX 5.6.0.7 software version, Hologic Horizon A, Waltham, MA). For each scan, participants wore light clothing and were asked to remove all materials that could attenuate the X-ray beam, including jewellery items and underwear containing wire. The coefficient of variation was less than < 3% for all measurements of segmental and whole-body body composition, including bone mineral density (g·cm^−2^), mineral content (g), FM (%), FM (g), FFM (g), and total body mass (g). The DXA was calibrated with phantoms according to the manufacturer’s guidelines each day before assessment.

#### Muscle thickness

Muscle thickness testing was carried out using b-mode ultrasound on the same day as DXA, with participants having refrained from training for at least 2 days. A single experienced investigator, blinded to participant allocation, conducted the ultrasound measurements. Ultrasonography used a wall-tracking ultrasound system (Samsung HS40, South Korea) with a 12 MHz linear array transducer to measure the thickness of the rectus femoris (RF) muscle. To ensure accurate measurements, the transducer was positioned perpendicular to the longitudinal axis of the thigh, with ample contact gel applied and minimal pressure exerted to prevent muscle compression (de Bruin et al. 1997; Seymour et al. 2009). The RF thickness was measured at 50% of the distance between the lateral epicondyle and the greater trochanter of the femur. Measurements were taken on both legs of each participant while lying supine, with both knees extended and relaxed and the feet maintained in a neutral position. Five images were captured for each measurement and the average value of these measurements was used as the RF thickness for further analysis (Thomaes et al. 2012).

### Statistical analysis

All statistical analyses were performed using the Jamovi software package (The Jamovi Project, v.1.6.23.0; downloadable at https://www.jamovi.org). Normality was checked by the Shapiro–Wilk normality test. A repeated measures linear mixed model fitted with a restricted maximum likelihood method and unstructured covariates was used to compare outcomes (muscle thickness [mm] and lean muscle mass [g]) between time (pre and post) and conditions (cluster and traditional). In addition, the same statistical treatment was performed to calculate the statistical differences in training volume between groups and between sessions, comparing the total training volume of each session with session 1 for each group. The effect size (ES) was calculated for interactions between conditions using Cohen’s d guidelines. Threshold values for ES were > 0.2 (small), > 0.6 (large), and > 2.0 (very large) (Hopkins et al. 2009). In addition, Pearson’s *r* was used to examine correlations between changes in muscle thickness and thigh lean mass from pre- to post-training for the participants under each condition. The level of significance for all tests was set to *α* = 0.05. Mean, 95% confidence intervals (CI), standard error (SE) and the t values were reported for all statistical analyses. Furthermore, we investigated the reliability of the ultrasound measurements by calculating intraclass correlation coefficients (ICCs) and standard errors of measurement (SEMs), with 95% CI (ICC = 0.970, SEM = 3.63%).

## Results

Of the 13 participants who originally volunteered to take part in the study, 3 dropped out during data collection. Two participants were excluded because they did not adhere to the nutritional protocol and the other dropped out due to personal reasons. Thus, we analyzed data from the 10 participants who satisfactorily completed the training protocol.

**Fig. 3:**
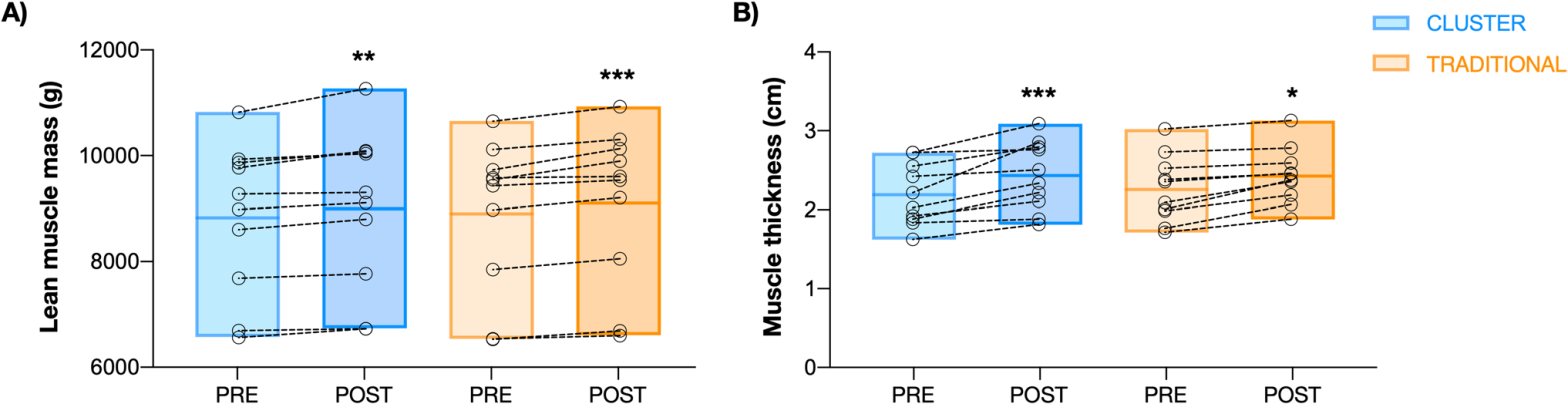
Mean and SD of lean mass (**A**) and rectus femoris muscle thickness (**B**) pre- and post-intervention for the CS condition (blue) and TS condition (orange). *Significant differences (*p* < 0.05) between pre and post (colour figure online)

Regarding lean tissue mass, statistically significant effects were only observed for time (*p* < 0.001, F = 44.3). No statistically significant interactions were observed between time and condition (*p* = 0.272, F = 0.27). As shown in Fig. 3A, post hoc analysis showed statistically significant increases in lean mass after the training intervention in both CS (mean [95% CI, SE, *p*, t]: 171.8 g [95% CI 88.5–255.2; SE = 39.7, *p* = 0.002, *t* = 4.33]) and TS (201.1 g [95% CI 117.7–284.5; SE = 39.7, *p* < 0.001, t = 5.1]), with a similar effect size (ES = 0.11 and ES = 0.13, respectively). No statistical pre (*p* = 0.652) or post (*p* = 0.254) intervention differences were observed between conditions.

Similarly, statistical analysis revealed that muscle thickness only showed statistically significant effects for time (*p* < 0.001, F = 38.1) with no statistical interactions observed between time and condition (*p* = 0.297, F = 1.15). As shown in Fig. 3B, post hoc analysis showed statistically significant increases in muscle thickness in both CS (mean [95% CI, SE, *p*, *t*]: 0.24 cm [95% CI 0.14–0.34; SE = 0.05, *p* < 0.001, *t* = 5.12]) and TS (0.17 cm [95% CI 0.07–0.27; SE = 0.17, *p* = 0.012, *t* = 3.6]). No statistical pre (*p* = 0.727) or post (*p* = 0.969) intervention differences were observed between conditions, with similar ES shown for both CS (ES = 0.56) and TS (ES = 0.42). In addition, no statistically significant (*p* > 0.05) correlations were found between changes in muscle thickness and lean mass for both the CS (R = 0.009) and TS (R = 0.506) groups (Supplementary Figure 5).

**Fig. 4:**
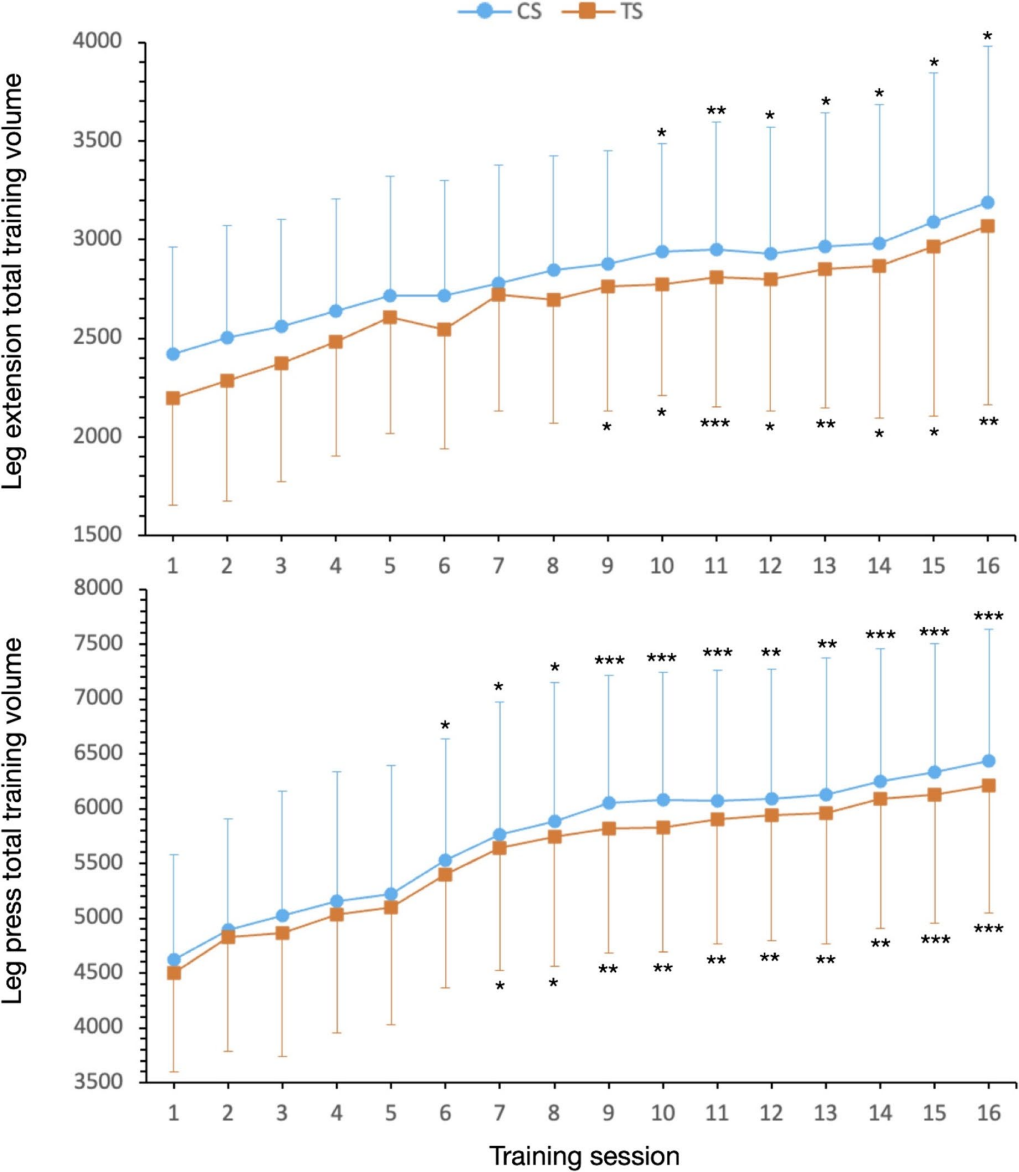
Mean ± SD of total session training volume for each condition. *p < 0.05, significant difference from session 1; **p < 0.01; ***p < 0.001

Regarding total training volume, no statistically significant differences (*p* > 0.05) were found between conditions (i.e., cluster vs. traditional) in any session for either the leg extension or leg press exercises (see Fig. 4). However, when comparing total training volume across sessions, a significant effect of the session was observed for both the leg extension and leg press exercises (*p* < 0.001, F = 24.9 and F = 22.6, respectively). Post hoc analyses further revealed significant increases in total training volume starting from sessions 7–8 when comparing the training volume of each session to that of the first session in both exercises and conditions (Fig. 4).

## Discussion

This study aimed to assess the effects of a CS protocol compared to a TS protocol on muscle thickness and FFM while equalizing volume by adjusting loads within the range of repetitions used. Results revealed significant increases in FFM within both conditions, with no statistical differences observed between the two protocols. Measures of muscle thickness of the RF showed similar improvements in both conditions as well. In addition, no statistically significant correlations were found between the muscle thickness gains and FFM increases for any condition. The cluster set method, combined with an effort intensity of 0–1 RIR within each cluster, proved to be effective in maintaining a high training intensity (i.e., % 1-RM) and promoting muscle hypertrophy. Alternatively, CS did not induce greater muscular gains compared to traditional training.

Current evidence indicates that volume is a primary programming variable for optimizing muscle hypertrophy (Figueiredo et al. 2018; Schoenfeld et al. 2017), with sets performed to or near failure (0–4 reps in reserve) (Helms et al. 2016). Likewise, it has been established that a wide range of repetitions (6–30) generates similar whole-muscle hypertrophy (Vargas et al. 2019; Schoenfeld et al. 2021). In this context, it has been proposed that the quantification of the target volume can be achieved by considering the total number of sets performed for each muscle group (Baz-Valle et al. 2021). However, previous studies on cluster training have employed designs where volume load (i.e., sets x repetitions x load) was equalized to a TS protocol (Jukic et al. 2020; Erick Carlos da Cunha Totó et al. 2019). Thus, previous studies in which CS and TS were compared assumed that volume load was equalized when the number of sets and repetitions and intensity (load in kg) was the same between conditions (Arazi et al. 2018; Goto et al. 2005). For example, when a load of 10 RM is used for both protocols, the TS condition will perform 10 RM while the CS condition will perform 5 repetitions and another 5 repetitions after 30 s. So, clusters of 5 repetitions, with loads equivalent to a 10 RM, would be quite far from failure (RIR-5, approximately), especially in the first block (Iglesias-Soler et al. 2016).

Furthermore, CS training protocols often employ higher intensities of load (based on %1-RM), which may not be most conducive to promoting muscle hypertrophy. For example, when a volume-matched 1-RM protocol is performed with loads equivalent to 10 RM, the TS condition will perform 4 sets of 10 repetitions with 60% of the 1-RM with a 2-min break while the CS condition will perform 8 sets of 5 repetitions with 75% of the 1-RM, with a 60-s break (Oliver et al. 2013). It could be argued that the protocol used in this example, is more resemblant of a traditional set protocol of 5 repetitions than a CS protocol. Hence, the use of individualized RIR for each cluster within a strength training set can be an alternative strategy during CS training. The RIR method is based on determining the number of repetitions an individual can perform before reaching the point of momentary muscular failure. RIR is expressed as a number indicating how many repetitions are left in reserve (Helms et al. 2016). Farinas et al., used a load of 10 maximum repetitions for a protocol of 30 repetitions, where an intra-series pause of 18.5 s was taken between each repetition (Farinas et al. 2021). Given the loads employed in these protocols, the repetitions performed would be considerably far from a muscular failure. While this may increase volume due to the higher number of repetitions that could be performed, using sets this far from failure may not optimize muscle hypertrophy (Schoenfeld and Grgic 2019a, b). Consequently, TS protocols might be more advantageous under these circumstances.

In this study, we equated the total number of sets on each limb (10 sets × 2 times/week), and the number of repetitions per set in each exercise (12 repetitions in TS and 4 + 4 + 4 repetitions = 12 repetitions in CS); however, the intensity of load (% 1-RM) was adjusted based on the number of repetitions performed. Specifically, the loads were individually modified so participants performed 12 repetitions and 4 repetitions with a RIR of 0–1 for TS and CS, respectively. Presumably, the total tonnage performed in the CS condition should have been higher than that in the TS condition. However, when evaluating the progression of loads over the 8-week protocol, no statistically significant differences were found between the two conditions (see  Fig. 4).

The joint and neural stress generated in the CS protocol may be one of the indicators that the total volume was not appreciably greater at the end of the 8-week study period. It is not comparable to evaluate the volume load in an individual session, where the CS protocol is necessarily greater than to maintain the greater stress for 8 weeks. For example, Iglesias-Soler et al. reported a much higher increase in volume load in the CS protocol (Iglesias-Soler et al. 2014). However, the modified variable in this study was not the load, but rather the greater number of repetitions, since the load was kept constant. In addition, the volume load was evaluated acutely, in one session, as opposed to longitudinally in our study over 8 weeks.

In our research, the progressive increase in loads was maintained with higher repetition ranges in the traditional group, 12 repetitions. However, in the 4-rep ranges, the increase in load diminished and generated lower increases in progression, possibly because it was closer to their maximum capacity to generate force. It is worth noting that 3 blocks of 4 repetitions at RIR 0–1 for such a long duration may increase the risk of injury in older participants; the average age of participants in our study was 21 years.

It is worth noting that the 40% increase in volume load can be considered a large effect. The threshold for achieving further strength gains diminishes progressively as an individual becomes more trained, particularly over a relatively short investigation period as implemented in the present study. It is reasonable to speculate that such large effects would not be feasible in advanced athletes or bodybuilders. Further research is warranted on well-trained individuals.

Our study had several noteworthy limitations. First, although DXA is regarded as a valid method for estimating lean mass (Guglielmi et al. 2016), it cannot provide isolated muscle volume analysis or distinguish between different muscle groups (Maroto-Izquierdo et al. 2022). DXA is only able to quantify the lean tissue mass of a region of the body. Additionally, DXA-derived lean mass measurements can be influenced by factors such as fluid content and changes in muscle water content, which can impact X-ray attenuation and potentially limit the accuracy of quantifying muscle-specific gains (Maroto-Izquierdo et al. 2022). It is important to acknowledge these limitations when using DXA as a tool for inferring changes in muscle mass. Second, although we measured changes in muscle thickness, which is considered a more valid method for assessing changes in skeletal muscle size compared to DXA, we only conducted measurements at the midpoint of the RF muscle. Evidence indicates that muscles may hypertrophy in a non-uniform manner (Wakahara 2015), and thus we cannot rule out the possibility that hypertrophic adaptations may have diverged at other sites along the RF. Moreover, we cannot necessarily extrapolate findings to other muscles of the quadriceps (i.e., vasti muscles) or in other body regions. Third, the study duration was limited to 8 weeks with twice-weekly training sessions. While significant improvements in muscle hypertrophy were observed within this timeframe, it would be valuable to investigate adaptations over longer training periods to assess whether results may ultimately diverge between conditions. Finally, the study sample consisted of individuals who were homogeneous in terms of age and training experience. Therefore, the generalizability of the results to different populations remains uncertain. Future research should consider incorporating these design elements to provide a more comprehensive understanding of the implications of various training approaches.

## Conclusions

The results of this study indicate that both CS and TS protocols promote statistically significant increases in lean tissue mass and muscle thickness over an 8-week training program in resistance-trained individuals. However, no statistically significant differences were observed between the two conditions, suggesting that they were equally effective in promoting muscle development. The effect sizes for lean tissue mass and muscle thickness were similar between the CS and TS conditions, indicating comparable magnitudes of improvement.

The findings also revealed no statistically significant differences in total weekly training volume between the CS and TS conditions for the leg press and leg extension exercises. It is widely known that the use of cluster training may offer an alternative approach to traditional resistance training, allowing for greater training volume within a session while maintaining the kinematic characteristics of the movement throughout each set.

## Supplementary Information

Below is the link to the electronic supplementary material.

Supplementary file1 (JPEG 144 KB) Correlations between fat-free mass and muscle thickness

## Abbreviations

BMI: Body mass index
CS: Cluster sets
CI: Confidence intervals
DXA: Dual-energy x-ray absorptiometry
ES: Effect size
FM: Fat mass
FMM: Fat-free mass
ICCs: Correlation coefficients
LE: Leg extension
LP: Leg press
MRI: Magnetic resonance imaging
RM: Maximum repetition
RF: Rectus femoris
RIR: Repetition in reserve
SE: Standard error
TS: Traditional sets

## References

[CR1] Arazi H, Khanmohammadi A, Asadi A, Haff GG (2018) The effect of resistance training set configuration on strength, power, and hormonal adaptation in female volleyball players. Appl Physiol Nutr Metab 43(2):154–164. 10.1139/apnm-2017-0327 29017017 10.1139/apnm-2017-0327

[CR2] Asadi A, Ramirez-Campillo R (2016) Effects of cluster vs. traditional plyometric training sets on maximal-intensity exercise performance. Medicina (Kaunas) 52(1):41–45. 10.1016/j.medici.2016.01.001 26987499 10.1016/j.medici.2016.01.001

[CR3] Baz-Valle E, Fontes-Villalba M, Santos-Concejero J (2021) Total number of sets as a training volume quantification method for muscle hypertrophy: a systematic review. J Strength Cond Res 35(3):870–878. 10.1519/JSC.0000000000002776 30063555 10.1519/JSC.0000000000002776

[CR4] Bonilla DA, Kreider RB, Petro JL, Romance R, Garcia-Sillero M, Benitez-Porres J, Vargas-Molina S (2021) Creatine enhances the effects of cluster-set resistance training on lower-limb body composition and strength in resistance-trained men: a pilot study. Nutrients. 10.3390/nu13072303 34371813 10.3390/nu13072303 PMC8308441

[CR5] Carneiro MA, de Oliveira JG, de Sousa J, Santagnello S, Souza M, Orsatti F (2019) Effects of cluster training sets on muscle power and force–velocity relationship in postmenopausal women. Sport Sci Health. 10.1007/s11332-019-00599-1

[CR6] Davies TB, Halaki M, Orr R, Mitchell L, Helms ER, Clarke J, Hackett DA (2022) Effect of set-structure on upper-body muscular hypertrophy and performance in recreationally-trained male and female. J Strength Cond Res 36(8):2176–2185. 10.1519/JSC.0000000000003971 35916746 10.1519/JSC.0000000000003971

[CR7] de Bruin PF, Ueki J, Watson A, Pride NB (1997) Size and strength of the respiratory and quadriceps muscles in patients with chronic asthma. Eur Respir J 10(1):59–64. 10.1183/09031936.97.10010059 9032493 10.1183/09031936.97.10010059

[CR8] Erick Carlos da Cunha Totó, Miguel Soares Conceição, Amilton Vieira, Fernando Pareja-Blanco, Martim Bottaro, Daniel Boullosa (2019) Are cluster sets an effective method to induce muscular hypertrophy in response to resistance training? Revista Brasileira de Ciencias do Sporte 10.1590/rbce.42.2019.071

[CR9] Farinas J, Mayo X, Giraldez-Garcia MA, Carballeira E, Fernandez-Del-Olmo M, Rial-Vazquez J, Kingsley JD, Iglesias-Soler E (2021) Set configuration in strength training programs modulates the cross education phenomenon. J Strength Cond Res 35(9):2414–2420. 10.1519/JSC.0000000000003189 31136543 10.1519/JSC.0000000000003189

[CR10] Figueiredo VC, de Salles BF, Trajano GS (2018) Volume for muscle hypertrophy and health outcomes: the most effective variable in resistance training. Sports Med (Auckland, NZ) 48(3):499–505. 10.1007/s40279-017-0793-0 10.1007/s40279-017-0793-0 29022275

[CR11] Franchi MV, Longo S, Mallinson J, Quinlan JI, Taylor T, Greenhaff PL, Narici MV (2018) Muscle thickness correlates to muscle cross-sectional area in the assessment of strength training-induced hypertrophy. Scand J Med Sci Sports 28(3):846–853. 10.1111/sms.12961 28805932 10.1111/sms.12961 PMC5873262

[CR12] Garcia-Ramos A, Gonzalez-Hernandez JM, Banos-Pelegrin E, Castano-Zambudio A, Capelo-Ramirez F, Boullosa D, Haff GG, Jimenez-Reyes P (2020) Mechanical and metabolic responses to traditional and cluster set configurations in the bench press exercise. J Strength Cond Res 34(3):663–670. 10.1519/JSC.0000000000002301 29076963 10.1519/JSC.0000000000002301

[CR13] Goto K, Ishii N, Kizuka T, Takamatsu K (2005) The impact of metabolic stress on hormonal responses and muscular adaptations. Med Sci Sports Exerc 37(6):955–963 15947720

[CR14] Guglielmi G, Ponti F, Agostini M, Amadori M, Battista G, Bazzocchi A (2016) The role of DXA in sarcopenia. Aging Clin Exp Res 28(6):1047–1060. 10.1007/s40520-016-0589-3 27256078 10.1007/s40520-016-0589-3

[CR16] Helms ER, Cronin J, Storey A, Zourdos MC (2016) Application of the repetitions in reserve-based rating of perceived exertion scale for resistance training. Strength Condition J 38(4):42–49. 10.1519/ssc.0000000000000218 10.1519/SSC.0000000000000218 PMC4961270 27531969

[CR17] Hopkins WG, Marshall SW, Batterham AM, Hanin J (2009) Progressive statistics for studies in sports medicine and exercise science. Med Sci Sports Exerc 41(1):3–13. 10.1249/MSS.0b013e31818cb278 19092709 10.1249/MSS.0b013e31818cb278

[CR18] Iglesias-Soler E, Carballeira E, Sanchez-Otero T, Mayo X, Fernandez-del-Olmo M (2014) Performance of maximum number of repetitions with cluster-set configuration. Int J Sports Physiol Perform 9(4):637–642. 10.1123/ijspp.2013-0246 24154989 10.1123/ijspp.2013-0246

[CR19] Iglesias-Soler E, Mayo X, Río-Rodríguez D, Carballeira E, Fariñas J, Fernández-Del-Olmo M (2016) Inter-repetition rest training and traditional set configuration produce similar strength gains without cortical adaptations. J Sports Sci 34(15):1473–1484. 10.1080/02640414.2015.1119299 26630355 10.1080/02640414.2015.1119299

[CR20] Jager R, Kerksick CM, Campbell BI, Cribb PJ, Wells SD, Skwiat TM, Purpura M, Ziegenfuss TN, Ferrando AA, Arent SM, Smith-Ryan AE, Stout JR, Arciero PJ, Ormsbee MJ, Taylor LW, Wilborn CD, Kalman DS, Kreider RB, Willoughby DS, Hoffman JR, Krzykowski JL, Antonio J (2017) International Society of Sports Nutrition Position Stand: protein and exercise. J Int Soc Sports Nutr 14:20. 10.1186/s12970-017-0177-8 28642676 10.1186/s12970-017-0177-8 PMC5477153

[CR21] Jukic I, Ramos AG, Helms ER, McGuigan MR, Tufano JJ (2020) Acute effects of cluster and rest redistribution set structures on mechanical, metabolic, and perceptual fatigue during and after resistance training: a systematic review and meta-analysis. Sports Med 50(12):2209–2236. 10.1007/s40279-020-01344-2 32901442 10.1007/s40279-020-01344-2

[CR22] MacInnis MJ, McGlory C, Gibala MJ, Phillips SM (2017) Investigating human skeletal muscle physiology with unilateral exercise models: when one limb is more powerful than two. Appl Physiol Nutr Metab 42(6):563–570. 10.1139/apnm-2016-0645 28177712 10.1139/apnm-2016-0645

[CR23] Maroto-Izquierdo S, Nosaka K, Blazevich AJ, Gonzalez-Gallego J, de Paz JA (2022) Cross-education effects of unilateral accentuated eccentric isoinertial resistance training on lean mass and function. Scand J Med Sci Sports 32(4):672–684. 10.1111/sms.14108 34851533 10.1111/sms.14108

[CR24] Morales-Artacho AJ, Padial P, Garcia-Ramos A, Perez-Castilla A (2018) Influence of a cluster set configuration on the adaptations to short-term power training. J Strength Cond Res 32(4):930–937. 10.1519/JSC.0000000000001925 29570595 10.1519/JSC.0000000000001925

[CR25] Moreno SD, Brown LE, Coburn JW, Judelson DA (2014) Effect of cluster sets on plyometric jump power. J Strength Cond Res 28(9):2424–2428. 10.1519/JSC.0000000000000585 24942176 10.1519/JSC.0000000000000585

[CR26] Nagatani T, Haff GG, Guppy SN, Kendall KL (2022) Practical application of traditional and cluster set configurations within a resistance training program. Strength Condition J 44(5):87–101. 10.1519/SSC.0000000000000700

[CR27] Oliver JM, Jagim AR, Sanchez AC, Mardock MA, Kelly KA, Meredith HJ, Smith GL, Greenwood M, Parker JL, Riechman SE, Fluckey JD, Crouse SF, Kreider RB (2013) Greater gains in strength and power with intraset rest intervals in hypertrophic training. J Strength Cond Res 27(11):3116–3131. 10.1519/JSC.0b013e3182891672 23736782 10.1519/JSC.0b013e3182891672

[CR28] Oliver JM, Kreutzer A, Jenke SC, Phillips MD, Mitchell JB, Jones MT (2016) Velocity drives greater power observed during back squat using cluster sets. J Strength Cond Res 30(1):235–243. 10.1519/JSC.0000000000001023 26121432 10.1519/JSC.0000000000001023

[CR29] Romance R, Vargas S, Espinar S, Petro JL, Bonilla DA, Schoenfeld BJ, Kreider RB, Benitez-Porres J (2019) Oral contraceptive use does not negatively affect body composition and strength adaptations in trained women. Int J Sports Med 40(13):842–849. 10.1055/a-0985-4373 31491790 10.1055/a-0985-4373

[CR30] Schoenfeld B, Grgic J (2019a) Does training to failure maximize muscle hypertrophy? Strength Condition J 41(5):108–113. 10.1519/SSC.0000000000000473

[CR31] Schoenfeld BJ, Grgic J (2019b) Does training to failure maximize muscle hypertrophy. Strength Condition J 41(5):108–113

[CR32] Schoenfeld BJ, Ogborn D, Krieger JW (2017) Dose-response relationship between weekly resistance training volume and increases in muscle mass: a systematic review and meta-analysis. J Sports Sci 35(11):1073–1082. 10.1080/02640414.2016.1210197 27433992 10.1080/02640414.2016.1210197

[CR33] Schoenfeld BJ, Grgic J, Van Every DW, Plotkin DL (2021) Loading recommendations for muscle strength, hypertrophy, and local endurance: a re-examination of the repetition continuum. Sports (Basel). 10.3390/sports9020032 33671664 10.3390/sports9020032 PMC7927075

[CR34] Seymour JM, Ward K, Sidhu PS, Puthucheary Z, Steier J, Jolley CJ, Rafferty G, Polkey MI, Moxham J (2009) Ultrasound measurement of rectus femoris cross-sectional area and the relationship with quadriceps strength in COPD. Thorax 64(5):418–423. 10.1136/thx.2008.103986 19158125 10.1136/thx.2008.103986

[CR35] Teixeira V, Voci SM, Mendes-Netto RS, da Silva DG (2018) The relative validity of a food record using the smartphone application MyFitnessPal. Nutr Diet 75(2):219–225. 10.1111/1747-0080.12401 29280547 10.1111/1747-0080.12401

[CR36] Thomaes T, Thomis M, Onkelinx S, Coudyzer W, Cornelissen V, Vanhees L (2012) Reliability and validity of the ultrasound technique to measure the rectus femoris muscle diameter in older CAD-patients. BMC Med Imaging 12:7. 10.1186/1471-2342-12-7 22471726 10.1186/1471-2342-12-7 PMC3342139

[CR37] Tufano JJ, Brown LE, Haff GG (2017a) Theoretical and practical aspects of different cluster set structures: a systematic review. J Strength Cond Res 31(3):848–867. 10.1519/JSC.0000000000001581 27465625 10.1519/JSC.0000000000001581

[CR38] Tufano JJ, Conlon JA, Nimphius S, Brown LE, Banyard HG, Williamson BD, Bishop LG, Hopper AJ, Haff GG (2017b) cluster sets: permitting greater mechanical stress without decreasing relative velocity. Int J Sports Physiol Perform 12(4):463–469. 10.1123/ijspp.2015-0738 27617387 10.1123/ijspp.2015-0738

[CR39] Vargas S, Petro JL, Romance R, Bonilla DA, Florido MA, Kreider RB, Schoenfeld BJ, Benitez-Porres J (2019) Comparison of changes in lean body mass with a strength- versus muscle endurance-based resistance training program. Eur J Appl Physiol 119(4):933–940. 10.1007/s00421-019-04082-0 30680448 10.1007/s00421-019-04082-0

[CR40] Vargas-Molina S, Romance A, Schoenfeld B, García-Sillero M, Petro J, Bonilla D, Kreider RB, Martín-Rivera F, Benítez-Porres J (2020) Effects of cluster training on body composition and strength in resistance-trained men. Isokinet Exerc Sci. 10.3233/IES-205122

[CR41] Vargas-Molina S, Petro JL, Bonilla DA, Baz-Valle E, Carbone L, Cannataro R, Benítez-Porres J (2021) Cluster sets for muscle hypertrophy: a short review. OBM Integrat Complement Med 10.21926/obm.icm.2201010

[CR42] Wakahara T (2015) Nonuniform muscle hypertrophy along the length induced by resistance training. Sports Perform. 10.1007/978-4-431-55315-1_14

[CR300] World Medical Association (2013) World medical association declaration of Helsinki: ethical principles for medical research involving human subjects. JAMA 310(20):2191–2194. 10.1001/jama.2013.281053 10.1001/jama.2013.281053 24141714

[CR43] Zhao X, Wang Z, Zhang J, Hua J, He W, Zhu S (2013) Estimation of total body skeletal muscle mass in Chinese adults: prediction model by dual-energy X-ray absorptiometry. PLoS One 8(1):e53561. 10.1371/journal.pone.0053561 23308254 10.1371/journal.pone.0053561 PMC3538629

