# Supplementary figures and images for: Cluster sets and traditional sets elicit similar muscular hypertrophy: a volume and effort-matched study in resistance-trained individuals
Source: Eur J Appl Physiol. 2025 Feb 11;125(6):1725–34. doi: 10.1007/s00421-025-05712-6 (PMC12174233; doi:10.1007/s00421-025-05712-6)

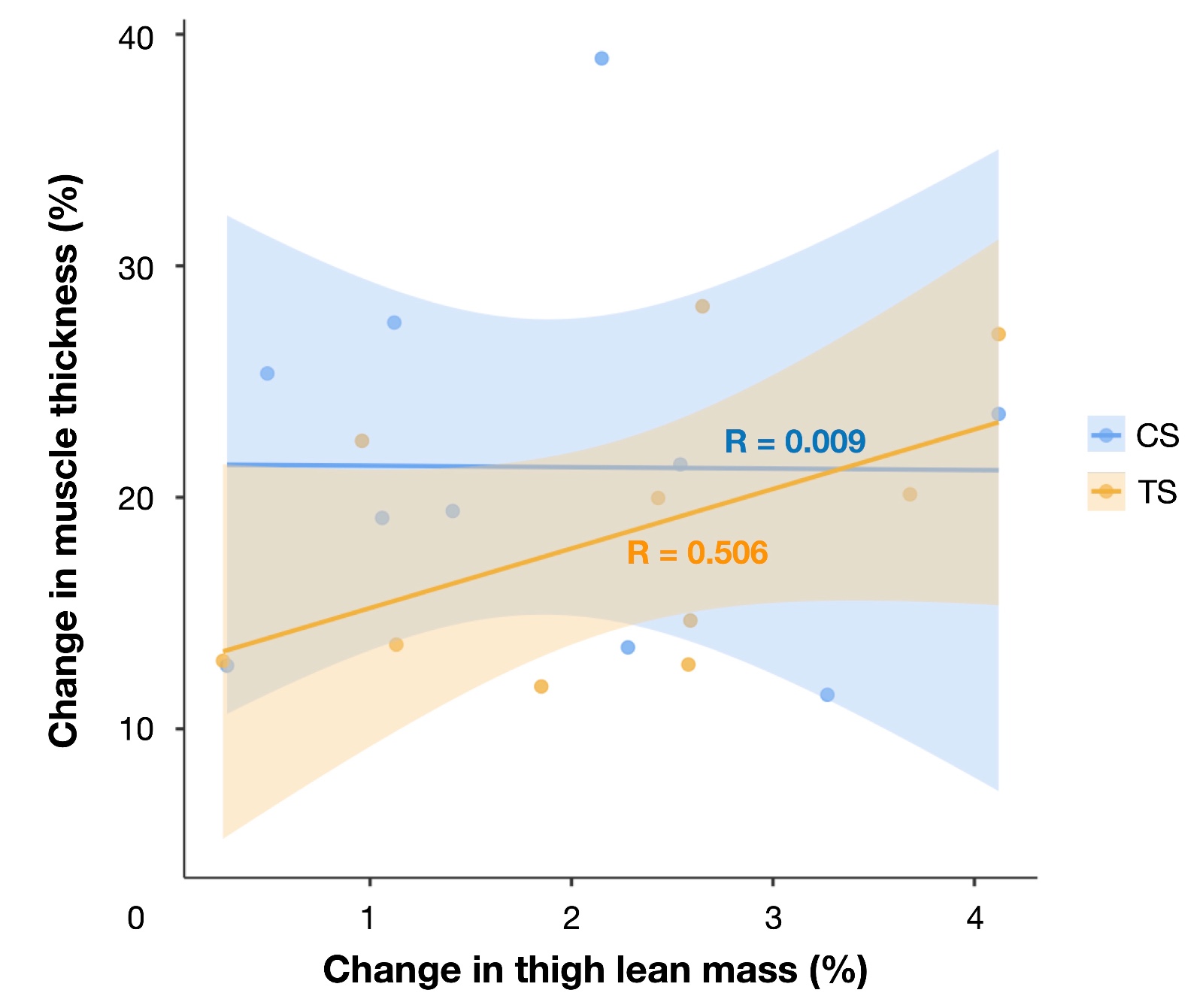

Supplement: Supplementary file 1 — Supplementary file1 (JPEG 144 KB) Correlations between fat-free mass and muscle thickness [file 421_2025_5712_MOESM1_ESM.jpeg]
